# NEWS

**DOI:** 10.7189/jogh.04-010203

**Published:** 2014-06

## DEMOGRAPHY

• The wealthy eastern province of Zhejiang is the first to implement a relaxation of China’s one–child policy, allowing more parents to have a second child. This is part of a plan to raise fertility rates and ease the financial burden on China’s ageing population. It allows couples to have two children if one of the parents is an only child; previously a couple could only have a second child if both parents were only children. Government figures showed that this policy, which covers 63% of the population, has averted 400 million births since 1980. (*Reuters,* 17 Jan 2014)

• Life expectancy in India has increased by five years in a decade, to stand at 67.3 years and 69.6 years for men and women, respectively. This increase is attributed to improved immunisation, nutrition, and prevention and treatment of infectious diseases. Maternal and infant mortality rates have fallen to 212 and 42 per 100 000, from 301 and 58 in the past decade. However, increasing life expectancy beyond 70 years depends on environmental factors (eg, clean drinking water) and better control of non–communicable diseases. Some experts sound a note of caution, noting that increased life expectancy would increase the disease burden. (*Times of India*, 29 Jan 2014)

• A recent study shows that the fertility rate of much of Africa is falling more slowly than expected, meaning that the continent’s overall population will rise sharply, its big cities will grow alarmingly, and there is an impending “youth bulge”. 78% of Africa’s people live in countries still transitioning to low mortality and fertility. There were 411 million children in 2010, with a predicted increase to 839 million in 2050. Although there will be lots of new entrants into the labour market, educating them will be expensive, and the continued reliance of precarious employment for young workers is worrying. It is argued that African governments must make more effort to spread the use of contraception, which is low by international standards. Without this, Africa risks having too many people with too few chances to escape poverty. (*The Economist*, 8 Mar 2014)

• China has revealed plans for state–led infrastructure construction, as it moves 100 million more people from rural areas to growing cities. The “National New–Type Urbanisation plan” details a massive building programme of transport networks, infrastructure and residential real estate, and China’s leaders hope that this will boost China’s flagging economic growth by boosting domestic demand. The plan aims to increase China’s urbanisation rate to 60% by 2020. However, this goal is complicated by China’s strict household registration system, which makes it difficult for those born in the countryside who move to the city to register for permanent resident status in the city. Therefore urbanisation can be reversed if rural migrants move back to their village upon losing their job. (*Financial Times*, 17 Mar 2014)

• Japan’s population fell by a record 244 000 people in 2013, accelerating a trend which began in 2004, and making it the fastest–ageing society on earth. There are warnings that the country’s population will fall from 127 million to 87 million by 2060, almost 40% of whom will be aged 65 or over. Mass immigration is one possible solution, but an estimated 650 000 immigrants are required each year – unprecedented in a largely homogenous society. Another solution is to boost fertility rates, which are amongst the world’s lowest. To succeed in this, Japan needs to make it easier for women to work, and address the country’s wide gender gap. Without such measures to boost its population, Japan’s already–high public debt will be further strained by the cost of caring for its elderly population, and it may have to adjust to having a reduced economic and political role in the world. (*The Economist*, 25 Mar 2014)

## ECONOMY

• The UN reported that the global economy will continue to grow over the next two years, but stronger international policy co–ordination is needed to steady residual fragility in the banking sector and geopolitics, which risk financial stability. Global economic growth is expected to be 3.0% in 2014 and 3.3% in 2015, compared to 2.1% in 2013. These forecasts are based on stronger–than–expected growth in the USA and an end to the Eurozone’s recession, although the eurozone will still experience high unemployment and austerity. Large emerging economies, such as China and India, have avoided deceleration. There are risks ahead, including misjudgements in ending the US Federal Reserve’s quantitative easing stimulus. Policy–makers need to balance an improved recovery with mitigating the effects of quantitative easing in major economies, plus advance reforms in the international financial system. (*UN News,* 18 Dec 2013)

• The world’s richest 85 people (who could all fit into a bus) have a combined wealth of US$ 1 trillion – as much as the world’s poorest 3.5 billion people, according to an Oxfam report; and the wealth of the 1% richest people is US$ 110 trillion, or 65 times as much as the poorest 50%. Oxfam is concerned that this concentration of resources threatens political stability and will increase social tensions. It fears that the wealthiest parts of the population will pass on their advantages to their children, and lock out others from equality of opportunity. It called on delegates at the 2014 World Economic Forum to tackle the problem, both by refraining from tax avoidance and using their wealth to seek political favors. (*The Guardian*, 20 Jan 2014)

• A study by the Center for Strategic and International Studies shows that private sector corruption in developing countries is a tax on growth that costs at least US$ 500 billion a year – more than triple all foreign aid in 2012. Corruption distorts marketplace incentives and creates economic inefficiencies, which are not directly measurable but weaken growth and undermine confidence in government. Bribe payers are more likely to be exposed, not the officials who accept bribes, thus perpetuating the problem. It calls for research on the impact of good governance on company value; the application of new technologies to expose corruption and policy changes; and compulsory anti–corruption components in free trade agreements. (*Thomson Reuters Foundation*, 22 Jan 2014)

• Sub–Saharan Africa is the world’ fastest–growing economy, and is undergoing a quiet manufacturing boom – almost always essential to development. Farming, services and exporting commodities still dominate, but new industries are emerging. Manufacturing’s share in GDP is steady at 10–14%. Industrial output is expanding as quickly as the rest of the economy, shown by the growth of domestic and overseas manufacturers. Many are benefiting from growth outside manufacturing, eg, retail. Construction booms foster access to high–voltage power, and the spread of mobile telephone services helps small suppliers, and there is growing local demand for African app and software developers. This is underpinned by improvements in education and human capital, and spurred by investment by Chinese workers. Many jobs could leave China for Africa if labour productivity continues to rise, and corruption and red tape are curbed. Africa is in a good position to industrialise, with favourable demography, urbanisation, an emerging middle class and strong services. (*The Economist*, 8 Feb 2014)

• Climbing out of, and staying out of, extreme poverty can be difficult; people can be driven back by unemployment, poor health and natural disasters, etc. A report by the Overseas Development Institute and the Chronic Poverty Advisory Network warns of poverty’s “revolving door”, and that progress in reducing extreme poverty may not continue. It calls for efforts to address the “three legs” of poverty: chronic poverty; becoming poor; and enabling those who have escaped poverty to keep moving up. It recommends three approaches to zero poverty: cash relief as a safety net; investment in education; and economic growth that helps the poorest by providing stable, safe and adequately paying jobs. It calls for more action on tackling chronic poverty, the addressing of which is expected in the post–2015 framework. (*Voice of America*, 10 Mar 2014)

## ENERGY

• The “MINT” countries of Mexico, Indonesia, Nigeria and Turkey are the focus of attention as the next global economic powerhouses. Common factors include large populations with a high percentage of working–age adults and geographical placements allowing them to take advantage of changing world trade patterns. Individually, these countries face problems with corruption, energy demands, infrastructure and the need for reform. Their governments are tackling these problems with enthusiasm and tenacity, leading to predictions that they could join the group of the world’s 10 largest economies within 30 years. (*BBC News,* 6 Jan 2014)

• Energy and water are crucial to human society and development. Both are tightly bound as water is needed for most energy generation; and the water sector needs energy to extract, treat and transport water. Both resources are under pressure as the global population grows towards 9 billion, leading to increases of an estimated 15% and 35% in water and energy consumption, respectively. Climate change means increased water variability and more extreme weather; decreasing water will make it harder to generate energy. To deal with these risks, the World Bank launched the *Thirsty Energy* initiative to help governments prepare for an uncertain future. “The water–energy interrelationship is critical to build resilience as well as efficient, clean energy systems. The time to act is now,” says Ms Rachel Kyte, World Bank Group Vice President and Special Envoy for Climate Change. (*World Bank,* 16 Jan 2014)

• *Business Insider* reported on Hans Rosling’s talk on energy inequality, which showed how 5 billion people still wash their clothes by hand. Hans Rosling believes that access to labour–saving devices like washing machines foster education and democracy. The middle chunk of 5 billion people (above the 2 billion of US$ 2/d, below the 1 billion of US$ 80/d) largely have access to electricity, but mainly wash their clothes by hand – time–consuming and hard work. He shows that the richest one billion people consume half of the world’s energy, and the poorest two billion one–sixth. Economic growth could allow everyone access to energy and labour–saving devices. He believes that wealthy populations cannot dictate others’ energy usage, and should concentrate on reducing their own energy consumption and implementing green energy. He showed the washing machine’s impact on his own family; it meant his mother could go to the library, read books to her son and learn English. (*Business Insider*, 29 Jan 2014)

• A team of researchers at the National Ignition Facility (NIF) at the Lawrence Livermore National Laboratory in California claim that for the first time, more energy has been extracted by controlled nuclear fusion than was absorbed by the fuel to trigger it. Although the gain was very small, this could still be a critical step towards ignition, the point beyond which more energy is generated than is put in, although fusion–energy generation is still a distant goal. (*Nature,* 12 Feb 2014)

• Achim Steiner, head of the UN Environmental Programme, warns that shale gas could become a liability in global efforts to limit climate change. Shale gas supporters state that it can help move countries away from carbon–intensive coal as it burns more cleanly than coal with less CO_2_ emissions. However, Achim Steiner believes that it will delay the vital transition from fossil to renewable fuels. Indeed, it could block progress towards low–carbon and zero–emission energy production by providing a distraction from this longer–term goal. (*The Guardian,* 26 Feb 2014)

## ENVIRONMENT

• The 2013 Annual Conference of the Society of Environmental Journalists examined the media role in an era of rapid climate change, and how to communicate the interconnected stories of population, development and environmental crisis. Journalists find the global media industry does not accept stories without established paradigms, and it is almost impossible to connect the science of climate change with the human impact of those changes. In response, journalists are working with researchers and activists who view environmental justice and population as inescapably linked. An example presented at the conference is the NGO Conservation through Public Health in Uganda working with remote communities in the Bwindi Impenetrable National Park to achieve both gorilla conservation and improved access to family planning. The initiative teaches people how to prevent human/ape disease transmission, and also deploys peer educators to spread information on contraception. (*Thomson Reuters Foundation*, 5 Oct 2013)

• The International Agency for Research on Cancer (IARC) declared that air pollution is a carcinogen, ranking it as the most important environmental carcinogen ahead of second–hand tobacco smoke. Its main risk is fine particles that can be deposited deep in the lungs. It is almost impossible to avoid air pollution, which may lead governments to introduce stricter controls. In 2010, there were more than 220 000 lung cancer deaths attributable to air pollution, and there is a link with a slightly increased risk of bladder cancer. Air pollution is a particular problem in China and India, and collective international action by governments is necessary to improve air quality. (*Associated Press*, 17 Oct 2013)

• High energy costs, declining competitiveness and on–going economic weakness are causing EU policy–makers to review Europe’s standards on climate–change regulation. These include tough rules on emissions and ensuring more use of renewable energy. However, the EU has now proposed an end to targets for renewable energy production, by introducing an overall goal that will probably be harder to enforce. It has reduced curbs on the environmental damage caused by fracking. Despite this, the EU introduced a 40% cut in carbon emissions by 2030. EU carbon emissions have already fallen sharply, but this is partly caused by contracting economic activity, and is subject to reversion. Environmental groups were critical of these announcements, describing them as “totally inadequate”, and of ignoring the costs of dealing with climate change. (*New York Times*, 22 Jan 2014)

• At the World Economic Forum in Davos, the OECD called for leaders to tackle the huge risk posed by carbon dioxide emissions. It recognised that although many countries have made progress, current pledges on reductions are not enough to maintain a 2°C ceiling on global temperature increases. Governments must implement plans for zero net emissions from fossil fuels from 2050 onwards. This is challenging as fossil fuels account for 67% of global electricity generation and 95% of transport energy. This is compounded by shale gas being a potential source of fossil fuels, investment in carbon–intensive technologies is more profitable than low–carbon technologies, and revenue from oil and gas contribute much to government revenue. The OECD calls for measures to deal with this by signaling increases in carbon prices such as reforming fossil fuel subsidies and addressing unclear and inconsistent policies. (*OECD*, 27 Jan 2014)

• The latest UN–backed report from the Intergovernmental Panel on Climate Change (IPCC) says that global warming is unequivocal, almost certainly due to human influence since the mid–20th century, and atmospheric concentrations of greenhouse gases – already at the highest levels for 800 000 years – will persist for centuries even if emissions stop. Continued emissions will cause further warming and changes in all components of the climate system, with dangers from the melting of the Greenland and Antarctic ice–sheets, rising ocean levels and more extreme weather events. “Limiting climate change will require substantial and sustained reductions of greenhouse gas emissions,” it states. (*UN News*, 30 Jan 2014)

## FOOD, WATER AND SANITATION

• November 19th is the UN’s World Toilet Day, which highlights the massive impact of inadequate sanitation on hundreds of millions of people world–wide. Described as “the biggest global development challenge of the 21st century”, one–in–three people lack access to a toilet, and diarrhoea is the world’s biggest killer of children under five. This is largely preventable, and the UN estimates a US$ 4 return for each US$ 1 invested in sanitation, giving children a better education and reduced pressure on health budgets. (*BBC News*, 19 Nov 2013)

• The World Bank has approved a US$ 500 million credit to improve piped water supplies and sanitation in the Indian states of Assam, Bihar, Jharkhand and Uttar Pradesh. Only 31% of the 167 million Indian rural households have access to tap water and domestic toilets, and 67% of the rural population practise open defecation. This fits with the government’s plan of ensuring that 90% of the rural population has access to piped water. The economic impact of inadequate sanitation is an estimated 6.4% of GDP. “Some 3.8 million women, who bear the burden of securing daily water supplies and dealing with illnesses from poor water and sanitation facilities, are expected to benefit from improved facilities that will be created in the project areas. The project will reduce the time spent by women in collecting water, which they can now use in other productive ways,” said Ms Onno Ruhl, the Bank’s country director for India. (*World Bank*, 7 Jan 2014)

• The UN Food and Agriculture Organisation (FAO) called for increased efforts to improve the operation of global food systems, stating that half of the world’s population is affected by under– and over–consumption of food. Despite abundant supplies, 840 million people go hungry each day, and the health of another 2 billion is compromised by nutrient deficiencies. Over–consumption increases the risk of diabetes, heart problems and other diseases. Looking ahead to 2050, when the world must support 9.6 billion people, there must be more emphasis on sustainably–produced nutrient–dense foodstuffs. Increased meat consumption means that sustainable livestock management is crucial. “If the global community invested US$ 1.2 billion per year for five years on reducing micronutrient deficiencies, the results would be better health, fewer child deaths and increased future earnings. It would generate annual gains of US$ 15 billion – a cost–to–benefit ratio of almost 13–to–1”, says Helena Semedo, the FAO Deputy Director–General. (*UN FAO*, 17 Jan 2014)

• The WHO urged a global drive against cancers linked to lifestyle – half of which are preventable – such as alcohol abuse, sugar consumption and obesity, as it estimated that new cases could increase by 70% to nearly 25 million a year over the next 20 years. It will be hugely expensive to treat these cases, so more emphasis must be placed on prevention and early detection. Low– and middle–income countries face the biggest burden due to increasing and ageing populations. Preventative measures include price increases for alcohol and sugary drinks, and government and society should support environments that enable healthy choices, eg, diet and exercise. (*The Guardian*, 3 Feb 2014)

• Globally, there is an increasing pressure on water supplies, due to agriculture, population and energy demands. Climate change is causing changing rainfall patterns, as the tropics and northern areas become wetter, and the already dry arid and semi–arid areas become drier. Scarce water resources could become a flash–point for conflict at both national and local scales. The international community has tool for de–escalating national conflicts, but local conflicts are harder to deal with and may be more critical. (*The Guardian*, 9 Feb 2014)

## PEACE AND HUMAN RIGHTS

• The Walk Free Foundation’s country–by–country survey on slavery found that more than 29 million people are living in slavery, and that 10 countries account for 70% of the world’s slaves. India has 14 million people enslaved, China 2.9 million and Pakistan 2 million. This fits with an International Labour Organisation survey, which estimated that 21 million people are in forced labour. The hidden nature of slavery means that data are difficult to collect and analyse, and some argue that emotive terms like slavery and forced labour can confuse the issue, and may risk limiting the choices of those in desperate circumstances. However, Mr Kevin Bales, the survey lead, believes that accurate labelling and tracking of slavery is the first step towards beating it. (*The Guardian*, 17 Oct 2013)

• The Dec 10 2013 International Human Rights Day was the 20th anniversary of the Vienna Declaration, which committed states to the promotion and protection of universal human rights and created the UN High Commissioner for Human Rights. The right to an education, the rights of children, the elimination of violence against women and the eradication of poverty were all envisaged in the declaration, but the attack on teen activist Malala Yousafzai shows the right to an education still cannot be taken for granted. Also, despite advances, Syria and the Central African Republic show that there are failings in protecting human rights. U.N. Assistant Secretary–General for Human Rights Mr Ivan Šimonović says that human rights abuses are often the first sign of conflict, and upfront action by the international community could prevent them. (*Voice of America,* 10 Dec 2013)

• The UN Secretary–General Ban Ki–moon spoke out about new anti–gay legislation in Nigeria, fearing that it may fuel prejudice and violence, whilst hoping that its constitutionality can be reviewed. It introduces a wide range of offences, including 14–year jail terms for cohabiting same–sex couples. It has drawn strong opposition from the UNHCR, UNAIDS and the Global Fund, by violating many human rights and jeopardising the HIV/AIDS epidemic response. Mr Ban has repeatedly called for the total decriminalisation of homosexuality, and for countries to ensure the protection of lesbian, gay, bisexual and transgender people. (*UN News*, 16 Jan 2014)

• Twenty years after a UN summit called for women to have more control over their lives, a new UN report found that today’s women have fewer children, are less likely to die in childbirth, literacy is higher, and the majority of countries have gender parity for primary education. However, progress has been limited in the poorest areas, with pregnancy and childbirth being the main cause of death in women aged 15–19. Women are paid less, are more often in unsecure employment, and there are gender gaps in secondary and tertiary education. Physical and sexual violence rates remain high; eg, 1–in–3 men in the DR Congo have been sexually violent. It highlighted growing economic inequality, and the increasing concentration of the world’s youngsters in poorer nations with less job prospects, and how their needs are central to their countries’ development agendas. (*New York Times,* 12 Feb 2014)

• UN members met for the annual discussion on women’s status, whilst facing calls to make gender equality a priority in the post–2015 framework. Progress on the female Millennium Development Goals (MDGs) has been disappointing, and countries affected by conflict and violence are furthest away from achieving them, as violence reduces women’s access to health care, welfare, economic opportunities and political participation. Gender and peace are linked: peace is vital for gender equality; and gender inequality can drive violence. A stand–alone goal on gender equality and women’s rights can contribute to peace, especially if it addresses attitudes and norms; and peace is vital to promote it. (*The Guardian*, 3 Mar 2014)

## SCIENCE AND TECHNOLOGY

• Research suggests that babies’ weak immune systems, which are vulnerable to bacterial infections, may be deliberately engineered by the body. This is indicated by high levels of red blood cells expressing the protein CD71, which suppress the body’s immune response. This allows beneficial microbes to colonise the baby’s gut, skin, mouth and lungs. This finding could lead to new treatments for infections in newborns. Also, temporarily reducing CD71 cells may improve vaccination uptake by allowing babies to be vaccinated at birth (rather than months afterwards) – the only time when many newborns in developing countries receive medical attention. (*Nature*, 6 Nov 2013)

• A study published in *The Lancet* costed health system strengthening and investment packages for maternal and newborn health, child health, immunisation, family planning HIV/AIDS and malaria, and modelled their health and socio-economic returns. It recognised that the substantial reductions in maternal and child deaths in the past 20 years are insufficient to achieve MDG 4 and 5. Inadequate health systems and inefficient use of limited resources means that the leading causes of maternal and child mortality are largely preventable. It found that an additional investment of US$ 5 per person/y in key countries would give a rate of return of up to 9 times. This represents an additional investment of US$ 30 billion a year, ie, a 2% increase above current spending rates. It would prevent 5 million maternal deaths, 147 million child deaths and 32 million stillbirths, and produce greater economic growth via improved productivity. (*The Lancet*, 19 Nov 2013)

• Mr Randy Schekman, a US biologist who won the 2013 Nobel prize in physiology or medicine, claims that leading academic journals are distorting the scientific process and represent a “tyranny” that must be broken. Pressure to publish in top–tier journals encourage researchers to cut corners and focus on “trendy” research rather than doing more important work. His laboratory will no longer send research papers to *Nature, Cell* and *Science*. He criticizes top–tier journals for artificially restricting the number of papers they accept, which stokes demand, and the widely–used impact factor, claiming that it distorts science results. Editors from some of top–tier journals defended their publications, eg, by saying that their mission was to serve science and scientists. (*The Guardian*, 9 Dec 2013)

• In 2012, over 500 potential cancer drugs were under investigation, more than five times as many as diabetes, the next biggest category. This is fuelled by increases in cancer cases as more people live longer; the rising price of cancer drugs; and the rapid expansion of scientific knowledge. All cancers arise from genetic changes within the patient’s cells, and understanding these can suggest ways of attacking them with tailored drugs. Although cancer can be caused by several mutations and new mutations can develop, DNA sequencing may lead to better understanding of these mutations and the development of ever–more tailored treatments. Patients could be offered new treatments once their cancer becomes resistant to a given treatment regime. Another approach is to prime immune cells to attack cancer cells, or to boost their activity. Eventually it may be possible to combine targeted drugs with immunotherapy. (*The Economist*, 4 Jan 2014)

• Researchers studying the Plague of Justinian, an outbreak of the bubonic plague in the 6th century that killed half the world’s population, have found that it was a different strain to the bubonic plague of the 14th century, which killed 50 million Europeans. This suggests that a new strain of the plague could emerge, if it follows the same pattern of separate strains of the bacterium evolving and infecting humans. However, today’s antibiotics can effectively treat the bubonic plague, reducing the risk of another large–scale pandemic. (*Skynews*, 28 Jan 2014)

## JOURNAL OF GLOBAL HEALTH's NEWS TEAM:

Rachael Artherton (editor), Lucinda Bell, Shweta Bhardwaj, Susanna Böling, Rachel Burge, Kevin C C Choy, Jenny Falconer, Holly Gardner, Chris Graham, Deirdrie Halligan, Katharine Heus, Nandita Kaza, Abigail King, Lois King, Stephen Malden, Simon McKenzie, Judith Oguguo, Ashley Parry, Ariane Renaud, Gregory Stimac, Yan Yu Tan, Ryan Wereski, Iwan Williams, Sandra Qi Wu

